# Supplementary material for: Strong high-energy exciton electroluminescence from the light holes of polytypic quantum dots
Source: Nat Commun. 2024 Jul 27;15:6334. doi: 10.1038/s41467-024-50432-8 (PMC11283451; doi:10.1038/s41467-024-50432-8)
Supplement: Supplementary file 1 — Supplementary Information [file 41467_2024_50432_MOESM1_ESM.pdf]

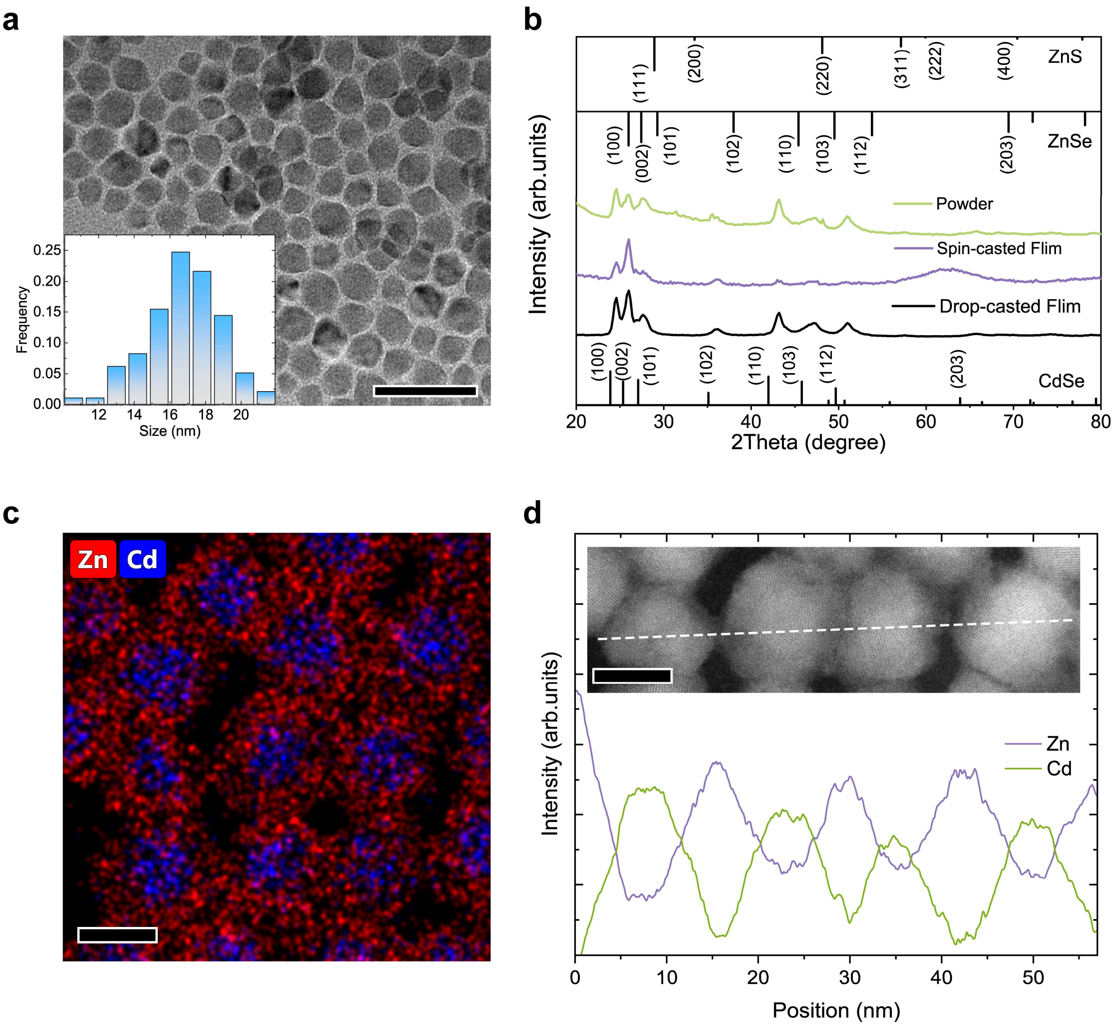

Supplementary Figure 1 | Transmission electron microscopy (TEM) image and The energy-dispersive spectroscopy (EDS) mappings of polytypic $Cd_xZn_{1-x}Se$-ZnS QDs. **a**, TEM image of QDs. A scale bar of 50 nm is also attached (black). Inset: Histogram of the particle size, with an average of 16.81 nm. **b**, X-ray diffraction (XRD) pattern of the powder and film samples of $Cd_xZn_{1-x}Se$ QDs. The bulk WZ CdSe, WZ ZnSe, and ZB ZnS powder XRD data (PCPDF: 08-0459, 15-0105, and 80-0020) are also provided for comparison. **c**, EDS elemental mapping of Cd and Zn elements from polytypic $Cd_xZn_{1-x}Se$-ZnS QDs. A scale bar of 10 nm is also attached (black). **d,** The line scanning of Cd and Zn elements from polytypic $Cd_xZn_{1-x}Se$-ZnS QDs. Inset: TEM of the scanned area. The dashed line represents the cross-section of scanning. A scale bar of 10 nm is also attached (black).

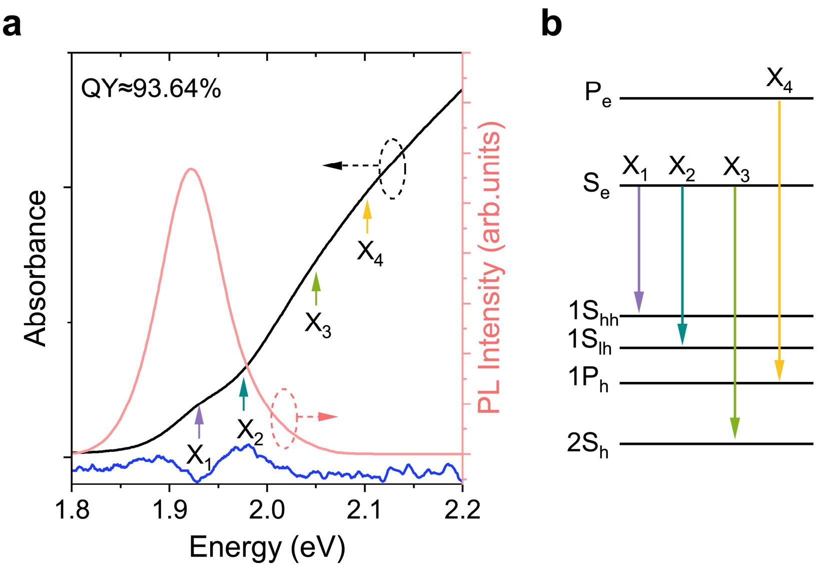

Supplementary Figure 2 | Excitonic structure of polytypic $Cd_xZn_{1-x}Se$-ZnS QDs. a, Absorbance (black) and photoluminescence (red) spectra of polytypic $Cd_xZn_{1-x}Se$-ZnS QDs. Different transition peaks are pointed out with different coloured arrows. The second derivative of the absorbance spectrum is also provided (blue). A quantum yield (QY) of ~93.6% is also noted. **b,** Energy levels of polytypic $Cd_xZn_{1-x}Se$-ZnS QDs. Transitions corresponding to the absorption peaks are noted. S_e and P_e indicate band-edge and higher-order electron states respectively; $1S_{hh}$, $1S_{lh}$, $1P_h$, and $2S_h$ indicate band-edge heavy hole state, light hole state, and two higher-order hole states respectively.

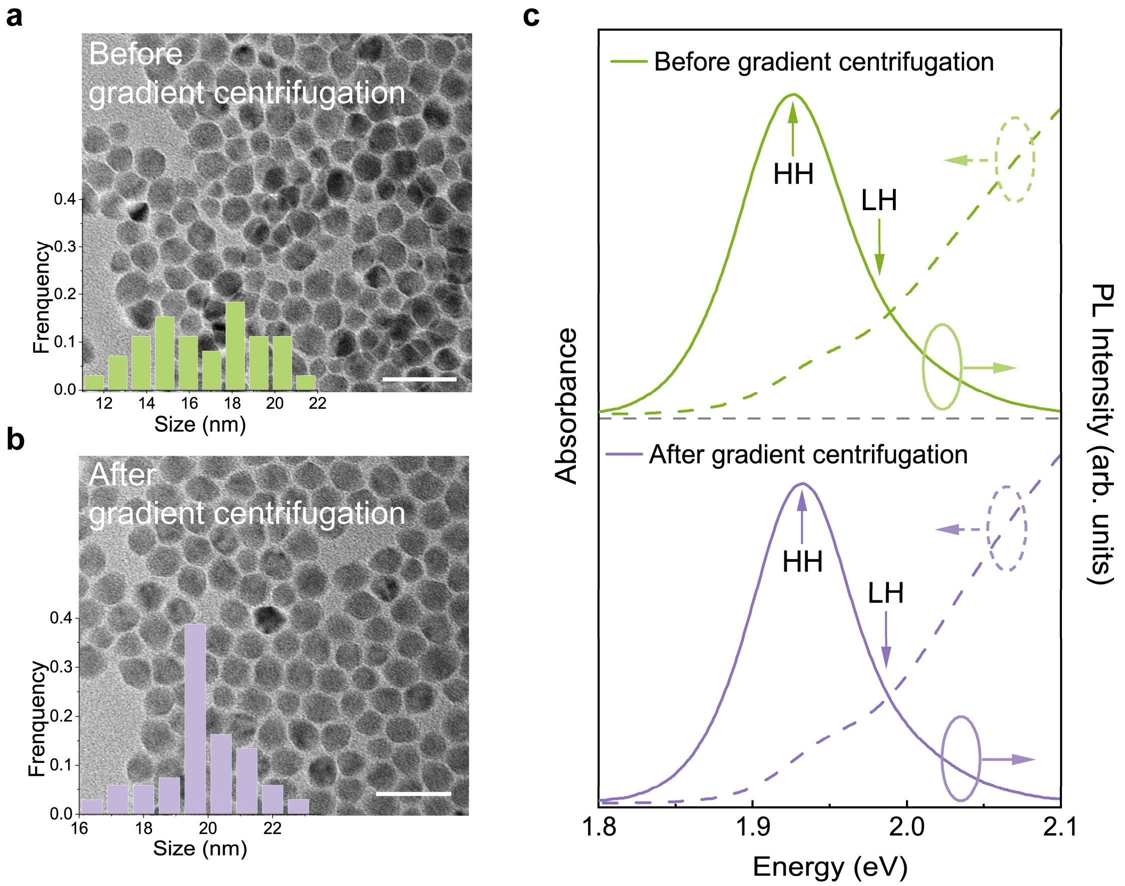

Supplementary Figure 3 | Gradient centrifugation of $Cd_xZn_{1-x}Se$-ZnS QDs. a,b, Transmission electron microscopy (TEM) image of $Cd_xZn_{1-x}Se$-ZnS QDs before (**a**) and after (**b**) gradient centrifugation. Inset: Histograms of the particle size. Scale bars of 50 nm are also attached (white). **c,** Photoluminescence (solid) and absorbance (dash) spectra of $Cd_xZn_{1-x}Se$-ZnS QDs before (green) and after (purple) gradient centrifugation. Changes in average size and size distribution of QDs result in slight differences in position and FWHM of the main peak, but the shoulder on the blue edge is almost unchanged after gradient centrifugation. HH: heavy hole; LH: light hole.

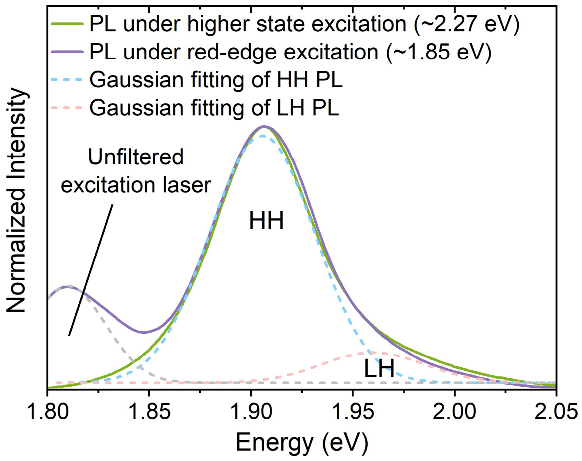

Supplementary Figure 4 | Normalized photoluminescence spectra of $Cd_xZn_{1-x}Se$ QDs under different excitations. High-energy photoluminescence shoulders can be observed under both red-edge (purple, solid) and higher-state (green, solid) excitations. The photoluminescence spectrum is fitted with two Gaussian peaks (blue and red, short dash), which are attributed to heavy-hole (HH) and light-hole (LH) photoluminescence, respectively. An additional peak observed on the red edge is the scattered excitation laser (grey, short dash).

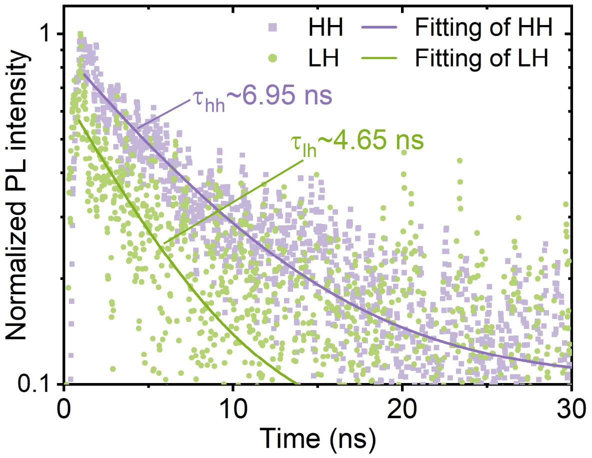

Supplementary Figure 5 | Time-resolved photoluminescence intensities of $Cd_xZn_{1-x}Se$ QDs in log coordinate. The decay of light hole (LH, green) and heavy hole (HH, purple) photoluminescence intensities are fitted with a single exponential. The lifetimes of LH and HH photoluminescence are given respectively.

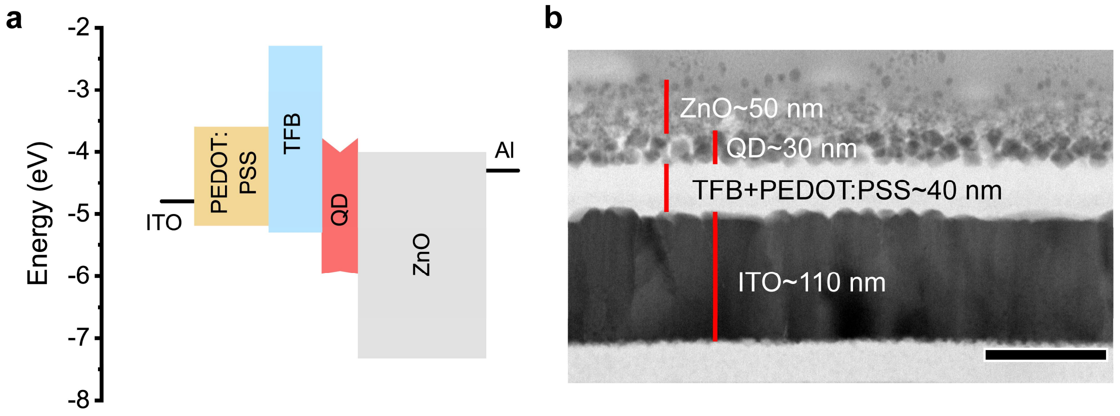

Supplementary Figure 6 | Structure of $Cd_xZn_{1-x}Se$-ZnS QD-LEDs. a, Schematic of the polytypic $Cd_xZn_{1-x}Se$-ZnS QD-LED structure, composed of ITO/PEDOT:PSS/TFB/QDs/ZnO/Al. **b,** Scanning electron microscopy (SEM) image of polytypic $Cd_xZn_{1-x}Se$-ZnS QD-LEDs. A scale bar of 100 nm is also attached (black). The thicknesses of different layers are noted with red lines. The boundary between TFB and PEDOT:PSS is unrecognizable.

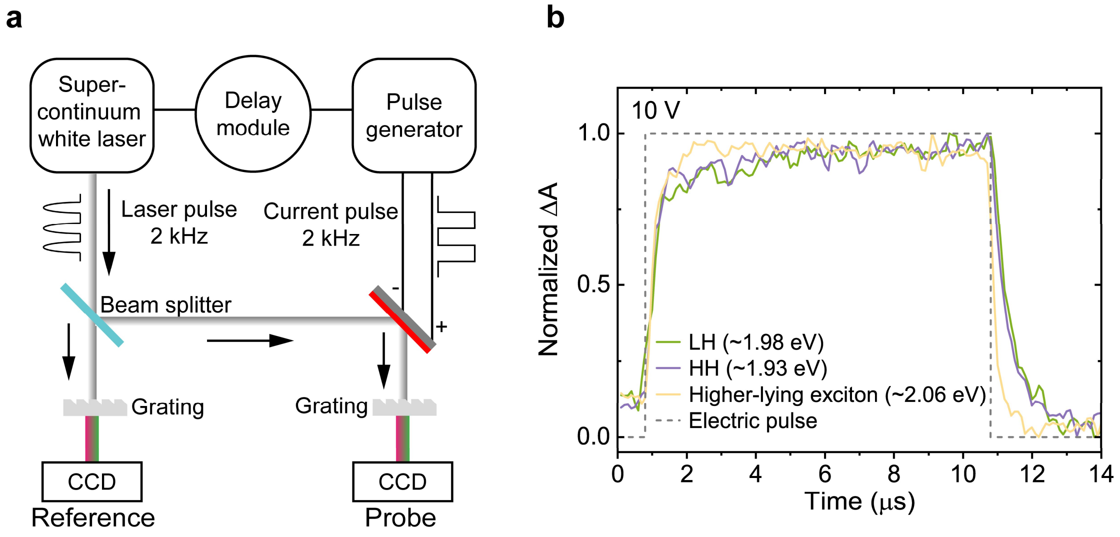

Supplementary Figure 7 | Electrically excited transient absorption spectroscopy (EETA) measurement. a, Schematic of EETA spectroscopy. The QD-LED is pumped with a current pulse (1 kHz, 10 μs), and a delayed pulsed supercontinuum white laser beam (2 kHz) is separated by a beam splitter, one of which is shined on the device, probing the change in transmission after excitation; the other is used as a reference signal to eliminate disturbances of laser intensity. **b,** Dynamics of LH (green), HH (purple), and higher-lying (yellow) excitons in electrically excited transient absorption spectra under 10 V bias. After the end of the current pulse (grey, dash), the carriers in QD-LEDs begin to relax, resulting in a decrease in the bleach of different excitons. The decay of LH and HH excitons is slower than higher-lying excitons, indicating a potential microsecond relaxation of higher-lying excitons to the LH/HH excitons. However, the decay of LH is as fast as HH, excluding the phonon bottleneck between LH and HH. This result is consistent with our conclusion in optically excited transient absorption spectra measurement.

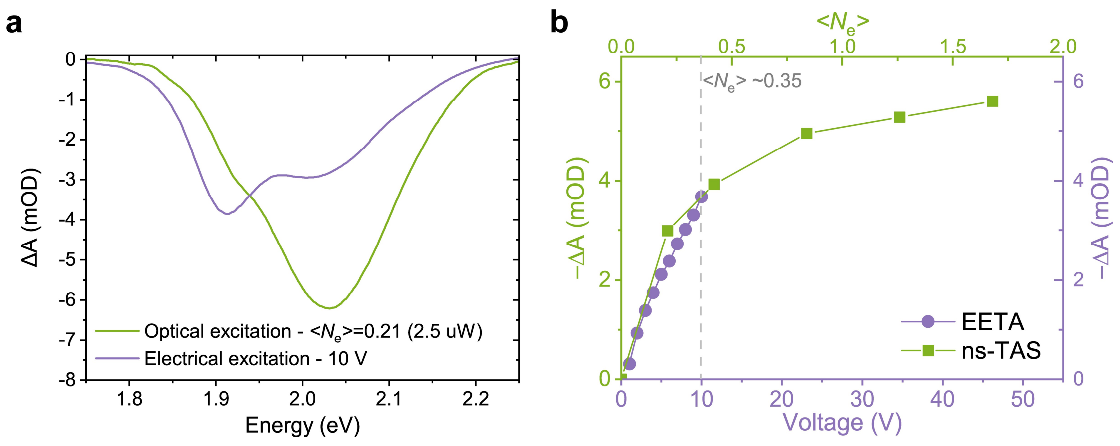

Supplementary Figure 8 | Calculation of averaged injected carrier. **a**, Optically excited nanosecond transient absorption spectrum (ns-TAS, green) and electrically excited transient absorption spectrum (EETA, purple) of the $Cd_xZn_{1-x}Se$-ZnS QD-LED. Averaged injected electrons under optical excitation can be calculated after measuring the power of excitation and absorption cross-section of the QD layer in the device. **b**, Comparison of the band-edge state bleaching in power-dependent ns-TAS (green) and voltage-dependent EETA (purple). When a 10 V voltage is applied to the QD-LED, the bleaching of the band-edge state in EETA is equivalent to the bleaching with an averaged injected electrons of ~0.35 per dot in ns-TAS of the same QD-LED.

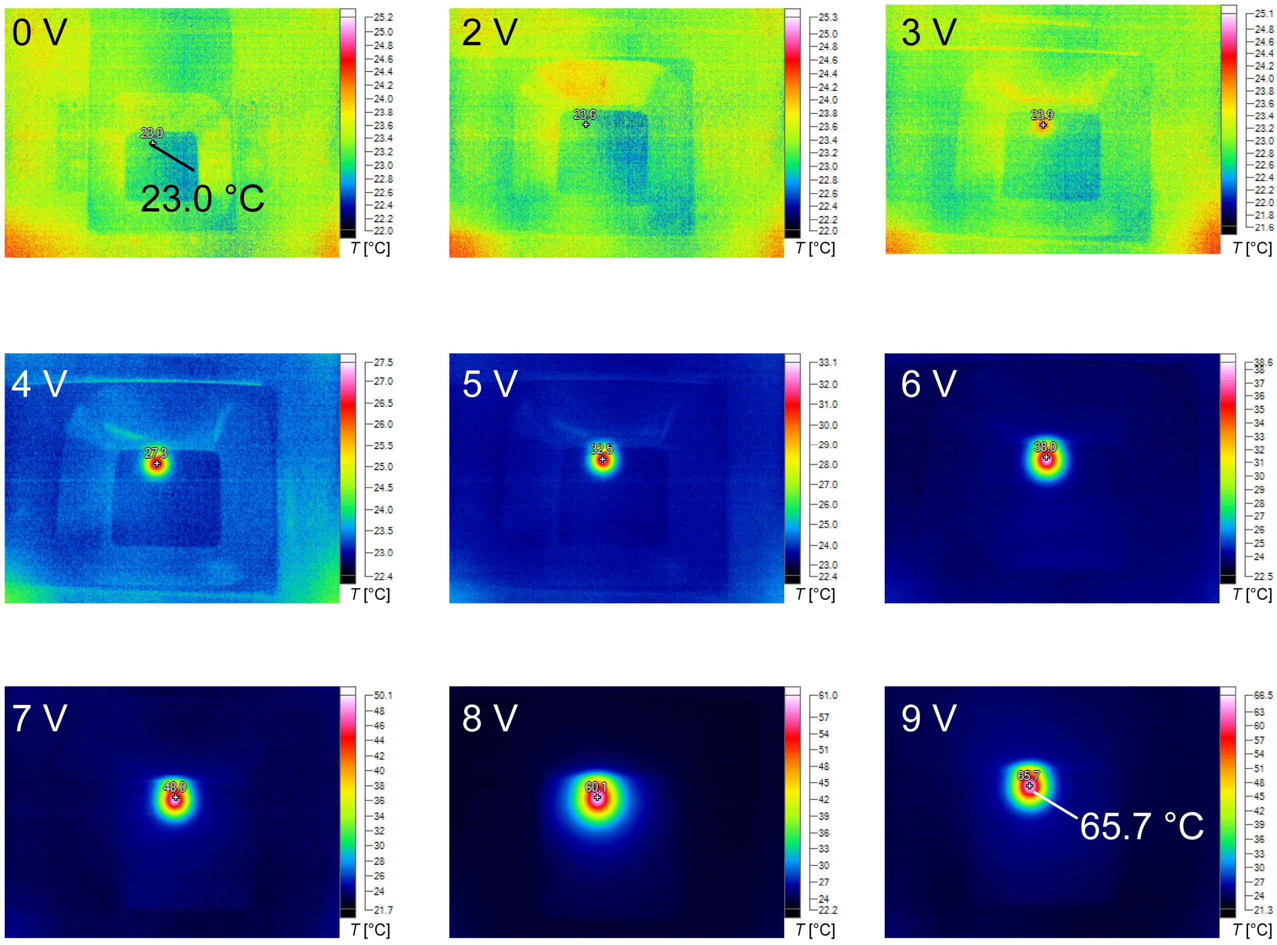

Supplementary Figure 9 | Device temperature under different biases. The temperature of our QD-LEDs continuously increases with external bias during our measurement. According to our calculation, the increase in temperature (from 23.0 °C to 65.7 °C) will result in an increased Boltzmann distribution of LH (~1.3 times, from 11.2% to 14.7%). This change in Boltzmann distribution is much smaller than the change in LH electroluminescence ratio (~3.6 times, from 22% to 80%), thus we only consider temperature as a minor attribution to the strong hot electroluminescence.

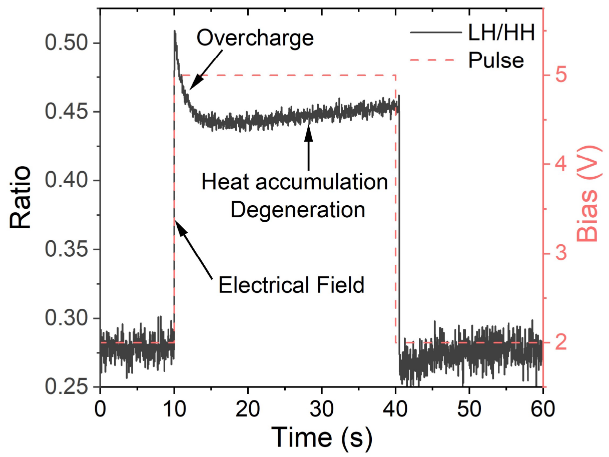

Supplementary Figure 10 | Time-dependent LH/HH electroluminescence ratio. A square pulse is used for excitation, whose high and low levels are 5 V and 2 V. As the bias increases, the ratio between light hole and heavy hole electroluminescence (LH/HH) rises immediately, much faster than the temperature change. Then other factors influence the ratio in a longer time scale, including overcharge, degeneration of LED, and heat accumulation. This result confirms our conclusion that temperature only plays a minor role in strong LH electroluminescence.

Supplementary Table 1| Theoretical calculation parameters. The shape of potential wells is supposed as trapezoidal, with band edges that transform from CdSe to ZnSe. Due to the lack of parameters, the effective mass of Cd-rich and Zn-rich parts are represented by the effective mass of CdSe and ZnSe respectively.

	m_e (m_0)	m_{hh} ($\mathrm{m_0}$)	m_{lh} (m_0)	CB offset (eV)	VB offset(eV)	Ref.
CdSe	0.13	0.45	0.16	—	—	1,2
ZnSe	0.21	0.60	0.145	0.85	0.23	3,4
ZnS	0.28	0.49		0.9	1.3	1

Supplementary References:

1. Haus, J. W., Zhou, H. S., Honma, I. & Komiyama, H. Quantum Confinement in Semiconductor Heterostructure Nanometer-Size Particles. *Phys. Rev. B* **47**, 1359-1365 (1993).

2. Bui, H., Karpulevich, A. & Bester, G. Excitonic fine structure of zinc-blende and wurtzite colloidal CdSe nanocrystals and comparison to effective mass results. *Phys. Rev. B.* **101**, 11 (2020).

3. Nabetani, Y., Ishibe, I., Sugiyama, K., Kato, T. & Matsumoto, T. Optical investigation of critical thickness and interface fluctuation in CdSe/ZnSe strained layer superlattices grown on InP. *Jpn. J. Appl. Phys. 1* **39,** 2541-2545 (2000).

4. Pandey, A. & Guyot-Sionnest, P. Intraband spectroscopy and band offsets of colloidal II-VI core/shell structures. *J. Chem. Phys.* **127**, 10 (2007).
